# The Relationship Between Reported Daily Nicotine Dose from NRT and Daily Cigarette Consumption in Pregnant Women Who Smoke in an Observational Cohort Study

**DOI:** 10.1093/ntr/ntad140

**Published:** 2023-08-03

**Authors:** Sophie Orton, Lisa Szatkowski, Felix Naughton, Tim Coleman, N-Ready team:, Miranda Clark, Sue Cooper, Anne Dickinson, Joanne Emery, Sarah Lewis, Lisa McDaid, Lucy Phillips, Ross Thomson

**Affiliations:** School of Medicine, University of Nottingham, Nottingham NG7 2RD, UK; School of Medicine, University of Nottingham, Nottingham NG7 2RD, UK; School of Health Sciences, University of East Anglia, NR4 7TJ, UK; School of Medicine, University of Nottingham, Nottingham NG7 2RD, UK; School of Medicine, University of Nottingham, Nottingham NG7 2RD, UK; School of Medicine, University of Nottingham, Nottingham NG7 2RD, UK; School of Medicine, University of Nottingham, Nottingham NG7 2RD, UK; School of Health Sciences, University of East Anglia, NR4 7TJ, UK; School of Medicine, University of Nottingham, Nottingham NG7 2RD, UK; School of Health Sciences, University of East Anglia, NR4 7TJ, UK; School of Medicine, University of Nottingham, Nottingham NG7 2RD, UK; School of Medicine, University of Nottingham, Nottingham NG7 2RD, UK

## Abstract

**Introduction:**

For nonpregnant people unable to quit smoking, the NHS recommends nicotine replacement therapy (NRT) for smoking reduction. This is not recommended during pregnancy due to concerns about higher nicotine intake than smoking alone. We investigated the relationship between daily nicotine dose from NRT and cigarette consumption reported by pregnant women receiving smoking cessation support.

**Methods:**

We conducted secondary analysis of data from currently smoking pregnant women, recruited from antenatal clinics (Nottingham University Hospitals, UK) or online between June 2019–September 2020. Participants set a quit date, received a prototype NRT adherence intervention, and reported cigarettes per day (CPD) and daily NRT dose (mg) via smartphone app for 28 days.

**Results:**

388 women were screened, 32 (8%) were eligible and joined the study. 24 (75%) submitted 510 app reports in total. 17 (71%) reported smoking and using NRT concurrently on at least one day, with concurrent use reported on 109 (21%) of app reports.

The relationship between daily NRT dose and CPD followed an exponential decay curve of approximately 7%. In multilevel repeated measures modelling using 4 linear splines (knots 17, 40, and 85 mg/NRT), significant fixed effects of daily NRT dose on CPD were observed for splines 1, 3, and 4. The strongest association was spline 1 (0–17 mg/NRT), where each 10 mg NRT increase was associated with a 0.6 CPD reduction (24% on average).

**Conclusions:**

Among women in a cessation study, many smoked and used NRT concurrently; within these women, daily nicotine dose and heaviness of smoking were inversely related.

**Implications:**

Findings have implications for the design of future interventions intended to reduce harm associated with smoking in pregnancy. They suggest using NRT alongside smoking in pregnancy could help some women reduce the number of cigarettes they smoke per day.

## Background

Smoking in pregnancy is a major public health problem; it is the biggest reversible cause of miscarriage, stillbirth, prematurity, low birth weight, perinatal, neonatal and sudden infant death and poorer infant cognition and behavioral outcomes.^1–3^ The prevalence of smoking in pregnancy is estimated to be between 13 and 39% in high income countries,^4–11^ and increasing in low- and middle-income countries.^12^ In England in 2020/2021, 9.5% of women were smoking at childbirth, with rates highest in economically deprived areas (Blackpool 21.4%).^13^ However, an estimated 23.3% of women in the UK smoke *at some point* during pregnancy,^14^ resulting in approximately 160,824 fetuses exposed to smoking in pregnancy annually,^15,16^ causing up to 5,000 miscarriages, 300 perinatal deaths and 2,200 premature births in the United Kingdom.^17^

The aim of the UK National Health Service (NHS) stop smoking support for pregnant women is complete abstinence; current guidelines state there is no safe level of smoking in pregnancy, and merely reducing smoking is discouraged.^18^ Pregnant women are only offered nicotine replacement therapy (NRT) if they are ready to quit smoking, and the NHS offers no further support to the 45% of women who smoke in pregnancy, but who do not make quit attempts.^19^ However, there is strong evidence that when most pregnant women cannot achieve abstinence, reducing smoking is very likely to be better for their and their babies’ health than “smoking as usual.” There are dose-dependent associations between heaviness of smoking and birthweight,^20^ low birth weight,^20–22^ increased risks of adverse pregnancy and adverse neonatal outcomes,^23^ and babies born to women smoking <10 cigarettes daily are heavier than babies born to women smoking >10 cigarettes daily.^20^ Helping pregnant women who cannot stop to instead reduce their smoking would substantially improve the health of up to 72,370 UK fetuses annually,^15,16,24^ and may lead to some women actually stopping smoking.

The National Institute for Health and Care Excellence (NICE) recommends NRT for reducing smoking to most people *who cannot stop.*^25^ This is because NRT used to cut down induces successful quit attempts and results in some stopping smoking, even though they did not set out to attempt cessation (relative risk for stopping smoking after using NRT to cut down, 1.87, 95% CI 1.43–2.44).^26^ Pregnant women are not recommended NRT for reducing smoking due to safety concerns. However, NRT could only be more harmful than smoking if nicotine alone causes most tobacco-related harms or generates higher nicotine concentrations than smoking. Both are highly unlikely; systematic reviews suggest using NRT instead of smoking is protective, not harmful, to the fetus,^27,28^ and pregnant women are exposed to less cotinine (primary nicotine metabolite) from NRT than when smoking.^29^ Nonpregnant people who not only smoke but also use NRT patches before quitting exhale less carbon monoxide (CO) and report smoking fewer cigarettes than when only smoking.^30^ Similarly, those using NRT to cut down reduced exhaled CO concentrations by 13–40%.^31^

*Pregnant women* who smoke and use NRT together behave similarly; compared to smoking alone, women on NRT patches who also used cigarettes smoked more lightly and exhaled less CO, but had similar cotinine concentrations.^32^ Those offered “dual” NRT for quitting (ie, two types of NRT together, at high dose), and reported some cigarette use rather than managing to stop smoking completely, smoked fewer daily cigarettes and exhaled less CO than when smoking, but salivary cotinine concentrations remained unchanged.^33^

From limited available evidence,^32,33^ it seems likely that in pregnancy, using NRT drives smoking reduction. However, we are not aware of any studies in pregnancy that have collected detailed data on use of NRT or that have investigated the association between NRT use and smoking using within-person methodology, and so no attempt has been made to address this important gap. We aimed to investigate the relationship between daily doses of nicotine from NRT and daily cigarette consumption in pregnant women.

### Aims

For pregnant women who have been offered NRT and are in the initial 28 days of a quit attempt to:

1. Describe reported daily nicotine doses from NRT use and daily cigarette consumption, including how these vary between women.
2. Investigate the within-person relationship between reported daily nicotine dose and daily cigarette consumption, including how this varies between women.

## Methods

### Design

This is a secondary analysis of data from two sequential cohort studies in which a prototype intervention intended to improve pregnant women’s NRT adherence was piloted. There was an earlier cohort, but data from this cohort could not be used because data on cigarettes smoked per day was not collected. The methods used to collect data are fully described in the study protocol.^34^

Ethical approval was given by the Nottingham 1 Research Ethics Committee (19/EM/0193). The paper is reported using the STROBE guideline for observational studies (S4).^35^

### Participants

Women were eligible to be included in the study if they were aged 16 or over and less than 25 weeks pregnant, smoked at least one cigarette daily (pre-pregnancy ≥10 cigarettes per day) and agreed to use NRT to try to stop smoking.

In cohort 1, eligible women were identified at Nottingham University Hospital Trust antenatal ultrasound and outpatient clinics. In cohort 2, due to COVID-19 restrictions, women were also identified through Facebook advertisements. Data were collected between June 2019–September 2020.

### Intervention

Participants received standard NHS smoking cessation care in pregnancy, during which women set a quit date within 14 days of the consultation. In addition to this, participants also received a counselling intervention, integrated into standard care, which involved being offered dual NRT, as a longer acting NRT patch (Nicorette 16-hour 15 mg or 25 mg; NiQuitin 24-hour 14 mg or 21 mg) with a fast-acting NRT (Nicorette Cools Lozenges (2 mg or 4 mg); Nicorette inhalator (15 mg) or Nicorette QuickMist mouth spray). Participants were advised to use as much NRT as required to ameliorate withdrawal symptoms, and not to stop NRT during smoking lapses of less than 14 days duration, provided they still aimed to stop smoking completely. Full details of the intervention have been described elsewhere.^34^

In cohort 1, the intervention was delivered during the face-to-face consultation with the researcher, following collection of baseline measurements. In cohort 2, due to COVID-19 restrictions, the intervention was delivered by a researcher via telephone at a separate appointment following collection of baseline measurements.

### Measures

#### Baseline:

Data were collected prior to intervention delivery when participants were still smoking. These included demographics such as, date of birth, ethnicity, educational qualifications, gestation, whether women had a partner who smoked, number of cigarettes smoked daily, use of e-cigarettes, smoking in previous pregnancies and how soon after waking women smoked their first cigarette. Saliva cotinine and exhaled air carbon monoxide (CO) concentrations were also collected at baseline.

In cohort 1, baseline measurements were taken in a face-to-face consultation. In cohort 2 baseline questions were asked via telephone and equipment sent to women via post for the remote collection of saliva and CO samples. In cohort 2, some participants declined to give an exact number of cigarettes smoked per day, and instead reported a range (eg, 8–10 cigarettes). For these participants, we used the upper estimate of their daily cigarettes per day.

#### Daily app reports (ecological momentary assessment (EMA) data):

After baseline procedures, participants downloaded a bespoke smartphone app called “NicUse”^36^ and, for 28 days, were asked to provide 1 app report per day about daily smoking and specific details of all NRT types used (type, doses and number of doses in 24 h) which were converted into a daily NRT dose (mg). Women were awarded £0.50 “credits” for each daily app report. They received an additional £1.50 for submitting seven consecutive app reports, and £5 for reporting on all requested 28 days (receiving up to a maximum of £25). These credits were then paid as shopping vouchers at the end of the study. Participants received daily prompts to complete NicUse surveys and, if a day’s survey was missed, it was possible to fill this in retrospectively but not more than 48 hours after intended.

## Analysis

All analyses were exploratory. Research aims and preliminary analysis plans were pre-registered^.37^ Statistical analysis was conducted using STATA V.17.^38^

Cohort characteristics, characteristics of those who completed app reports, and completeness of app reports are presented.

### Describing Patterns of, and Any Variation Between, Women’s Daily Reports of Nicotine Dose from NRT and Daily Cigarette Consumption

Descriptive statistics are presented to describe between-participant daily smoking behavior (proportion of days smoked; mean, median and range of cigarettes smoked per day), daily NRT use (proportion of days NRT used; mean, median and range of daily NRT dose) and dual smoking and NRT use (proportion of days dual use reported).

We present time series graphs showing within-participant cigarettes per day between quit date and 28 days later, and daily NRT dose between quit date and 28 days later (S1 and S2).

### Relationship Between Reported Daily Nicotine Dose from NRT and Daily Cigarette Consumption, and How This Varies Between Women

We used an exploratory approach to model the relationship between cigarettes per day and daily NRT dose. On visual examination of the plotted data it was evident that the between-participant univariable relationship was non-linear, instead it decayed exponentially. We therefore used exponential decay curve regression to model the data. We fitted a multilevel repeated measures model using the *mixed* command in STATA (level 1: daily measures, level 2: baseline time-invariant measures), using a simple linear spline to capture the non-linearity.^39^ We compared a model with 3 splines and 4 splines, using the AIC test to determine which model best fit the data. The final model had 3 knots set at equal percentiles of the data (17, 40, and 85 mg), creating 4 linear splines.

We examined an a priori determined interaction between baseline heaviness of smoking index (HSI, defined as “low,” “moderate” or “high”)^40^ and daily nicotine dose. This was not significant, and so was excluded from the final model.

We used an iterative approach to identify the following variables that were potential confounding factors to the association between daily nicotine dose and daily cigarette consumption: *days from quit date, e-cigarette use* (level 1)*, baseline HSI, ethnicity, highest education,* and *baseline partner smoking* (level 2). These variables were selected based on available data within the cohort, and informed by previous literature on factors associated with smoking in pregnancy.^41,42^ These were entered individually into the multilevel repeated measures model, and coefficients, *p*-values and 95% confidence intervals (CIs) calculated. None were significant in this univariable analysis at the *p* < .05 level. We then entered these variables consecutively, with those variables with univariable *p*-values closest to significant first, into a multivariable model to determine if any reached significance alongside other potential confounding variables. We also re-examined the *HSIxDaily NRT Dose* interaction to confirm nonsignificance alongside other potential confounders. None reached significance in multivariable analysis at the *p* < .05 level, and so all were omitted in the final model. We examined autocorrelation between the repeated measures using exponential residuals.^39^ These were significant using the likelihood ratio test, and so were retained in the final model.

We handled missing data using available case analysis.

## Results

### Cohort Characteristics

In cohort 1, 189 women were assessed for eligibility and 12 (6%) participated and, in cohort 2, 199 were assessed for eligibility and 20 (10%) participated. Main reasons for ineligibility included being a nonsmoker, already accessing stop smoking support, not being interested in participating and not willing to set a quit date and/or use NRT. In total, 32 women consented to join the study, and 24 participants completed app reports, with a total of 510 app reports across all participants. The mean number of days that app reports were completed was 21.5 (standard deviation [SD] 8.7, range 3–28) and median 25 (interquartile range 6).

The mean age of participants completing app reports was 29 years, and 100% were White British. 12% had no educational qualifications (no General Certificate of Secondary Education, GCSE’s, obtained age 16, or equivalent) and 50% were educated to GCSE-level. The average number of cigarettes smoked per day at baseline was 9.7, and most (71%) had a “low” HSI index at baseline. Full cohort characteristics are presented in Table 1.

**Table 1.** Participant characteristics

Characteristic	*N*(%) or mean/SD or median/IQR as appropriate All cohort (*N* = 32)	*N*(%) or mean/SD or median/IQR as appropriate Participants who completed daily app reports (*n* = 24)
**Age, mean (SD)**	28.9 (7.5)	29.3 (8.3)
**Ethnicity, *n* (%)**		
White British	30 (93.8)	24 (100)
**Qualifications, *n* (%)**		
None	4 (12.5)	3 (12)
GCSEs* or equivalent	15 (46.9)	12 (50)
A-Levels or equivalent	10 (31.3)	7 (29.2)
Degree or equivalent	3 (9.4)	2 (8.3)
**Gestation** (weeks + days), **mean (SD)**	14 weeks, 3.5 days (27.7 days)	14 weeks, 5 days (29.4 days)
**Partner who smokes**, *n* (%)		
No partner	6 (18.8)	4 (16.7)
Partner who smokes	20 (62.5)	15 (62.5)
Partner who is a non/ex-smoker	6 (18.8)	5 (20.8)
**Number of cigarettes smoked per day pre-pregnancy, mean (SD)**	17 (6.6)	16.1 (4.9)
**Smoking status in previous pregnancies, *n* (%)**		
No previous pregnancies	1 (3.1)	1 (4.2)
Yes	27 (84.4)	20 (83.3)
No	4 (12.5)	3 (12.5)
**Number of cigarettes smoked per day at baseline, mean (SD)**	10 (SD 5.1)	9.7 (SD 4.9)
**Heaviness of smoking index at baseline, *n* (%)**		
Low	22 (68.8)	17 (70.8)
Moderate	10 (31.3)	7 (29.2)
Heavy	0 (0)	0 (0)
**Saliva cotinine concentration baseline,** **ng/ml** (24 observations), **mean (SD)**	150.1 (82.9)	147.4 (82.8) (23 observations)
**Exhaled carbon monoxide concentration baseline, ppm** (21 observations), **mean (SD)**	16.8 (9.5)	16.6 (9.7) (20 observations)

*General Certificate of Secondary Education, obtained aged 16

E-cigarette use was low within the sample. Across the 510 app reports, e-cigarette use was reported on 7 (1.4%) days, by 3 participants (range 1–5 days e-cigarettes were used).

### Smoking behavior

Across the 510 app reports, smoking was reported by 17 participants (71%) on 166 days (33%). The mean number of days smoking was reported was 6.9 (SD 8.2, range 0–27), and on days when participants did report smoking the mean cigarettes per day was 4.2 (SD 2.7).

The within-person variance of cigarettes per day was 1.4 (SD 1.2). Time series graphs displaying the within-participant patterns of cigarettes per day between quit date and 28 days later can be seen in S1. Seven participants smoked zero cigarettes per day and maintained this for the duration of their app reports. Others smoked a small number of cigarettes (1–2) for < 7 days before managing to quit smoking. Three participants reported short lapses in their smoking, smoking 1 cigarette in one of their app reports, before successfully resuming their quit attempt. 10 participants showed daily fluctuations in their smoking, and some days reporting no smoking. Four of these participants frequently reported smoking >5 cigarettes per day.

### NRT Use

All participants completing app reports (*N* = 24) reported using NRT on at least 1 day. Across the 510 app reports, NRT use was reported on 440 days (86%). The mean number of days NRT was reported was 18.3 (SD 9.6, range 2–28). The mean daily NRT dose was 51.8 mg (SD 43.2, median 40 mg, range 0–160 mg).

There was substantial within-person variance of daily NRT dose (variance 224753.2, SD 96.8). Time series graphs displaying the within-participant patterns of daily NRT dose between quit date and 28 days later can be seen in S2. The level of NRT women reported using varied daily (Figure S2). 10 women used a low dose (<50 mg) NRT consistently throughout the 28 days, with minor fluctuations. 4 women used a consistently higher dose (>100 mg) consistently throughout the 28 days. Two women who reported low daily doses of NRT (approximately 25 mg) discontinued use within 3–4 days. Four women increased their daily NRT dose within the first seven days of initiating use, which may have been in response to cravings or advice from their stop smoking advisor.

### Dual Smoking and NRT Use

71% (17/24) of participants reported both smoking and using NRT on the same day for at least one day. Across the 510 app reports, dual use was reported on 109 days (21%).

### Relationship Between Daily Nicotine Dose and Daily Cigarette Consumption

The relationships between NRT dose and cigarettes per day for each individual is presented in Figure 2. Although there is individual variation in the relationship between daily nicotine dose and daily cigarette consumption, for some women there was a clearer relationship between higher daily NRT dose (mg) and smoking fewer cigarettes.

**Figure 1.**
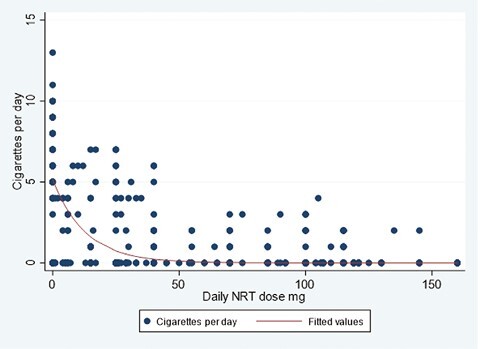
Relationship between cigarettes per day and daily NRT dose.

The between-person relationship between daily NRT dose and cigarettes per day followed an exponential decay curve (Figure 1), with a starting value of just over 5 (ie, women with an NRT dose of zero smoke on average 5 cigarettes per day). The rate of decay is approximately 7%, such that the fitted model indicated that women smoked no cigarettes per day at a daily NRT dose exceeding approximately 50 mg. There was, however, variability between women; the regression equation for the decay function accounts for approximately 60% of the explained variability in cigarettes per day.

**Figure 2.**
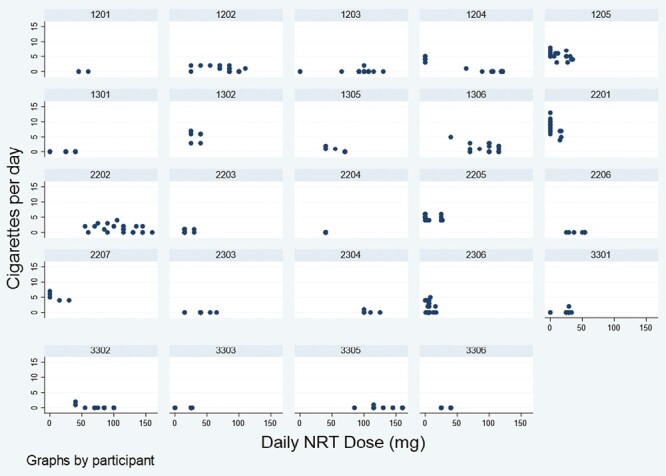
Within-person relationship between cigarettes per day and daily NRT dose (mg).

### Multilevel Model

**Figure 3.**
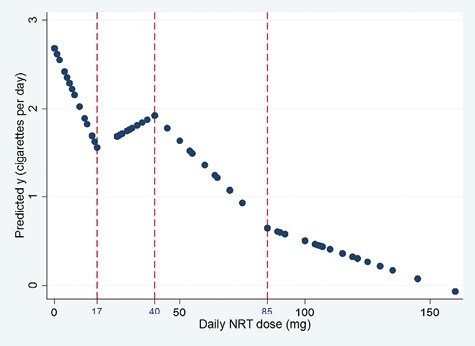
Predicted cigarettes per day in multilevel repeated measures model, controlling for participant as a random effect.

In the multilevel repeated measures model, knots were placed at equal percentiles of the data (17, 40, and 85 mg), creating 4 linear splines (Figure 3). The inclusion of individual participants as a random effect was found to significantly improve the model (*p* < .001) compared to a standard linear regression. Significant fixed effects of daily NRT dose (mg) were observed for splines 1, 3, and 4 (Supplementary File 3). The strongest association was observed for spline 1 (0–17 NRT mg), where each 1 mg increase in daily NRT dose was associated with a reduction of 0.06 cigarettes smoked per day (or in other terms, each 10 mg increase in NRT was associated with a reduction of 0.6 cigarettes per day). In spline 3 (40–85 NRT mg), each 1mg increase in daily NRT dose was associated with a reduction of 0.03 cigarettes per day, and in spline 4 (86–160 mg) each 1mg increase in daily NRT dose was associated with a reduction of 0.01 cigarettes per day.

## Discussion

Seven in ten pregnant women who were trying to quit smoking with NRT smoked and used NRT on the same day, and such dual use was reported in one fifth of women’s daily of app reports. When dual use was reported, there was generally an inverse relationship between daily nicotine dose from NRT and daily cigarette consumption. Modelling indicated that each 10mg increase in daily NRT dose was associated with a reduction of 0.6 cigarettes per day (24% reduction in cigarettes per day on average). For someone using 4–5 pieces of nicotine gum daily, this equates to a reduction in cigarettes per day of 40%.

A limitation of our study is the small sample size, which means that, whilst novel, our between-participant analyses is exploratory. A further potential limitation was our reliance on self-reported NRT dose and cigarette consumption. Self-reports of smoking rates in particular have been found to under-estimate actual smoking.^43^ Furthermore, as this study was a cessation study, participants may have felt biased towards reporting no smoking. However, if smoking were under-reported in our study, this would have diluted the relationship between NRT dose and cigarette consumption, but our bespoke NicUse app reports of cigarettes smoked per day have shown a strong relationship with exhaled CO concentrations.^33,36^ Reports of NRT use have been found to be more complete and accurate than retrospective questionnaire reports,^44^ so we can have confidence that our self-report measures reflect smoking and NRT use. A further potential limitation is the lack of ethnic diversity within our sample, who were all White British, however the sociodemographic profile of our sample was similar to other pregnancy cohorts, which have high proportions of women who were White British with generally lower education.^42,45,46^

A major strength of our study is its originality. This is the most detailed data on use of NRT during pregnancy and the relationship between daily NRT dose and cigarette consumption, using within-person data. Previous research has been limited to between-person investigations, which can obfuscate associations at the individual level and introduce ecological fallacy-type errors. Compared to retrospective reports, our EMA data and use of repeated measures of women’s NRT use and smoking behavior have good ecological validity, allowing for day-to-day fluctuations in behavior and minimizing recall bias.^47,48^ This study included only participants who agreed to use NRT to try and quit smoking, as this is how NRT is generally used in pregnancy, findings are likely to be applicable to usual clinical settings and use of NRT.

Few studies have investigated the relationship between heaviness of smoking and NRT use in pregnant women. One, a Cochrane review,^49^ did this in nonpregnant people. Four studies in this review found that people using NRT for smoking reduction were more likely to reduce their cigarettes per day by 50–75%; however, it was not possible to combine these studies and present a pooled estimate.^49^ We are only aware of two studies that have examined the relationship between heaviness of smoking and NRT use in pregnancy. In the first,^32^ pregnant women trying to stop smoking with the aid of NRT but having smoked at 2 weeks after their intended quit date reported smoking fewer cigarettes and had significantly lower exhaled CO levels, but there was no change in their salivary cotinine concentrations. Similarly, previous analyses of the current dataset^33^ found that on day 7 after women’s intended quit date, those who used NRT and smoked had significantly lower exhaled CO concentrations compared to pre-NRT exposure levels, but saliva cotinine concentrations were unchanged. Our study adds to this previous literature by using EMA data to provide granularity about how the daily relationship between NRT use and smoking in pregnancy varies.

One possible explanation for the observed inverse relationship between daily NRT use and smoking is nicotine self-titration. Research suggests that people modify their nicotine intake to maintain a steady blood nicotine (or cotinine) concentration, known as “nicotine self-titration.”^50^ Pregnant women may similarly self-titrate their nicotine intake such that blood nicotine concentrations are maintained at a stable level, presumably determined by their addiction. While it is possible that women in this study were titrating their nicotine intake by adjusting the way they smoke, for example, smoking fewer cigarettes but inhaling more deeply or increasing puff frequency,^51^ research using biomarkers of smoking suggests that this is unlikely. In these studies, in women who smoked and used NRT, salivary cotinine (a biomarker of nicotine indicating both smoking and NRT use) remained unchanged, whereas exhaled CO (a biomarker of smoking) significantly reduced.^32,33^ An alternative explanation for the relationship between daily nicotine dose and cigarette consumption could be day-to-day fluctuations in motivation; on days when women are particularly motivated to quit or cut down their smoking they may also feel more motivated to take their allocated NRT treatment and cut out cigarettes. For motivational fluctuation to explain study findings, women’s motivation to stop smoking would need to vary; however, we know little about the stability of motivation for cessation during gestation, and so future studies could include motivational measures within EMAs. Our findings have important implications for the design of future interventions intended to reduce harm associated with smoking in pregnancy. They provide reassurance that using NRT alongside smoking in pregnancy, for example in “preloading” or “cut down to quit” approaches is unlikely to result in increased nicotine exposure, but is likely to result in decreased exposure to other harmful toxicants within cigarette smoke.

## Conclusions

Among women enrolled into a cessation study, many smoked and used NRT concurrently; within those that did, daily nicotine dose and heaviness of smoking were inversely related. Our findings suggest using NRT alongside smoking during pregnancy could help some women reduce the number of cigarettes they smoke per day.

## Supplementary Material

A Contributorship Form detailing each author’s specific involvement with this content, as well as any supplementary data, are available online at https://academic.oup.com/ntr.

ntad140_suppl_Supplementary_File_S1

ntad140_suppl_Supplementary_File_S2

ntad140_suppl_Supplementary_File_S3

ntad140_suppl_Supplementary_File_S4

## Notes

Declarations of competing interest: The authors declare no competing interests.

Primary funding: This article presents independent research funded by the National Institute for Health Research (NIHR) under the Programme Grants for Applied Research programme RP-PG-0615-20003, and the National Institute for Health and Care Research (NIHR) School for Primary Care Research (project reference 524). TC is an NIHR Senior Investigator.

The views expressed are those of the author(s) and not necessarily those of the NIHR or the Department of Health and Social Care.

Data access requests can be made to Professor Tim Coleman.
