# Supplementary material for: The Relationship Between Reported Daily Nicotine Dose from NRT and Daily Cigarette Consumption in Pregnant Women Who Smoke in an Observational Cohort Study
Source: Nicotine Tob Res. 2023 Aug 3;26(2):212–9. doi: 10.1093/ntr/ntad140 (PMC10803113; doi:10.1093/ntr/ntad140)
Supplement: ntad140_suppl_Supplementary_File_S1 [file ntad140_suppl_supplementary_file_s1.pdf]

Supplementary file 1

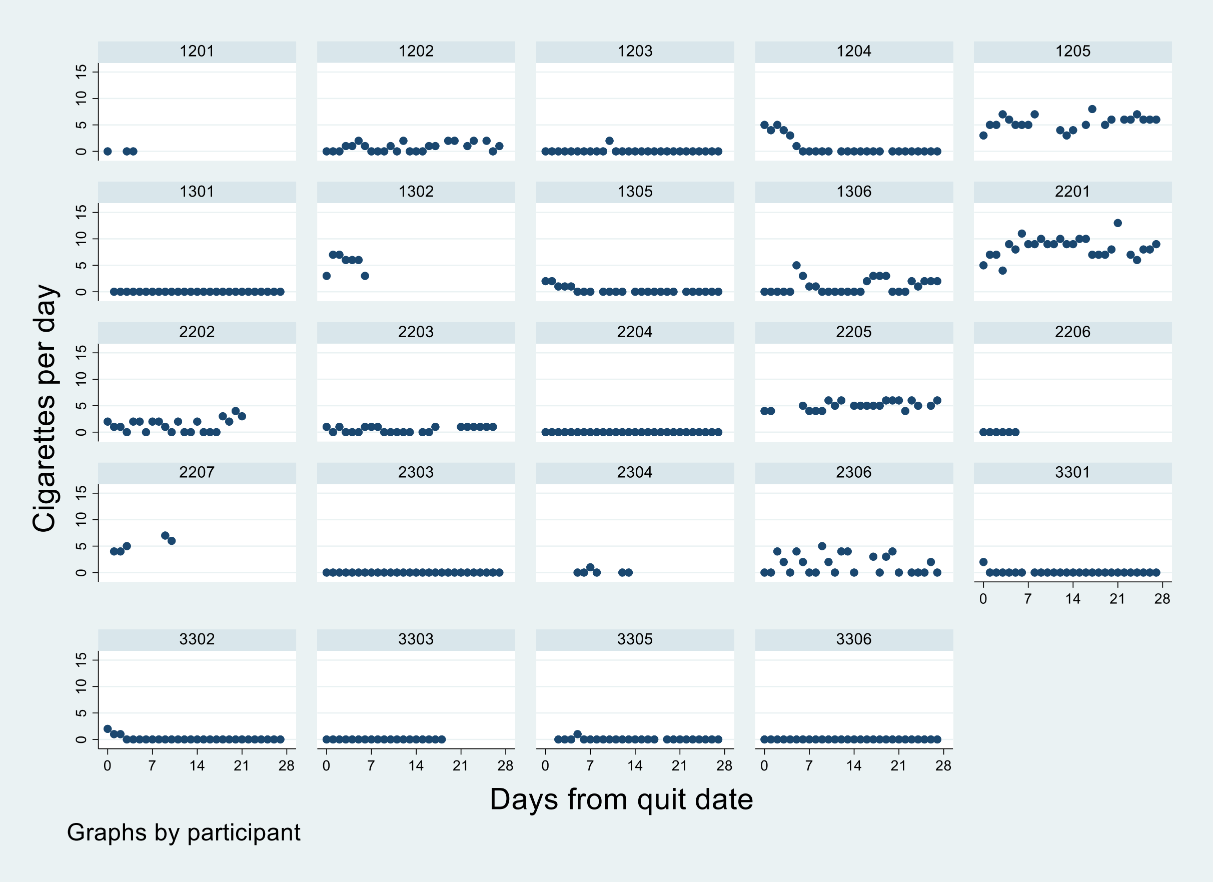

Figure S1. Cigarettes per day and days from quit date, each participant presented individually

Note: zero is no cigarettes smoked, missing data points are days where no app report was made

There was considerable variation in within-participant cigarettes smoked per day (figure S1). Seven participants smoked zero cigarettes per day and maintained this for the duration of their app reports. Others smoked a small number of cigarettes (1-2) for < 7 days before managing to quit smoking. Three participants reported short lapses in their smoking, smoking 1 cigarette in one of their app reports, before successfully resuming their quit attempt. 10 participants showed daily fluctuations in their smoking, and some days reporting no smoking. Four of these participants frequently reported smoking >5 cigarettes per day.
