# Supplementary material for: The Relationship Between Reported Daily Nicotine Dose from NRT and Daily Cigarette Consumption in Pregnant Women Who Smoke in an Observational Cohort Study
Source: Nicotine Tob Res. 2023 Aug 3;26(2):212–9. doi: 10.1093/ntr/ntad140 (PMC10803113; doi:10.1093/ntr/ntad140)
Supplement: ntad140_suppl_Supplementary_File_S2 [file ntad140_suppl_supplementary_file_s2.pdf]

Supplementary File 2

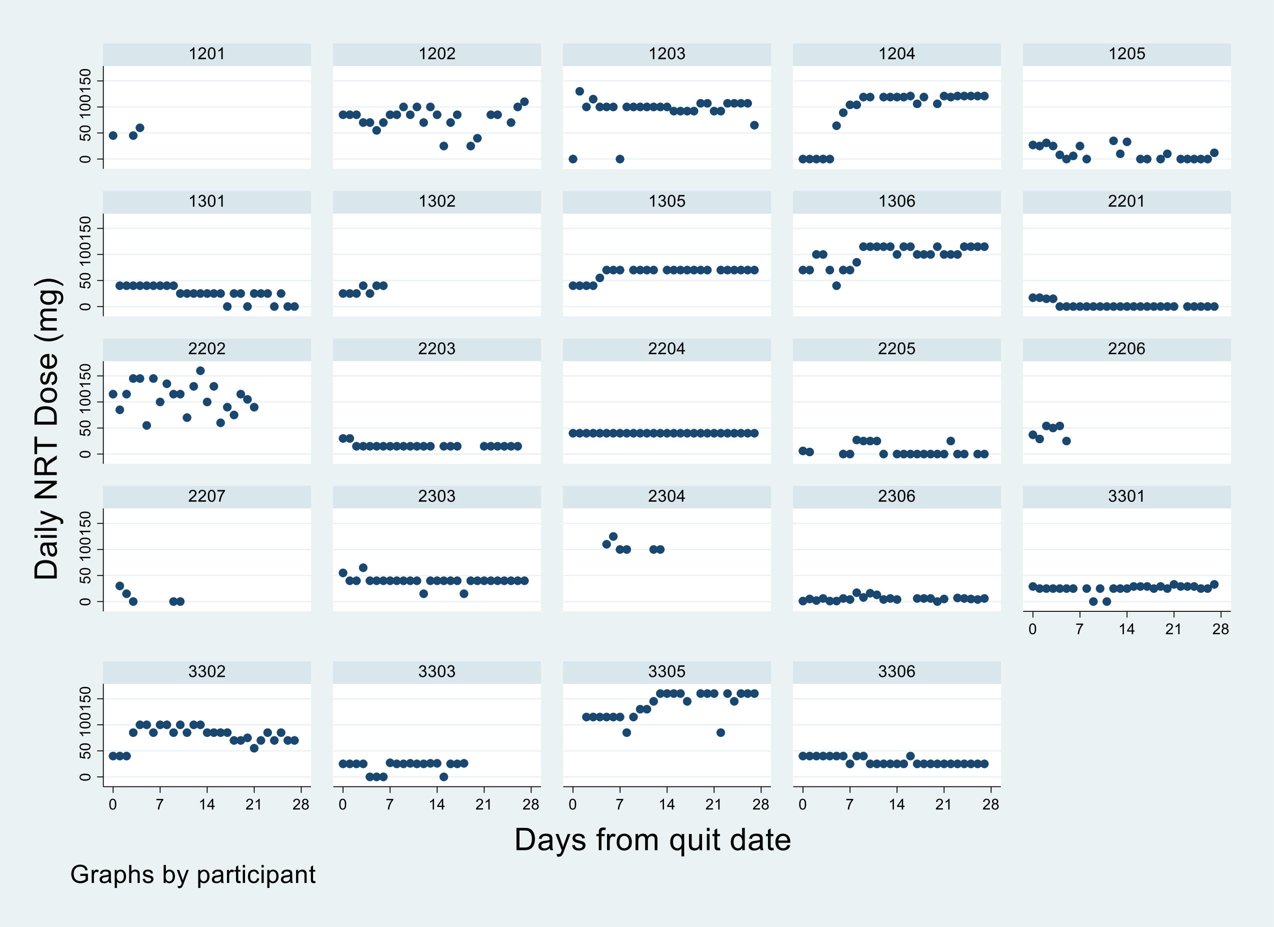

Figure S2 Daily NRT dose (mg) between quit date and 28 days later

Note: zero is no NRT use, missing data points are days where no app report was made

The level of NRT women reported using varied daily (figure S2). 10 women used a low dose (<50mg) NRT consistently throughout the 28 days, with minor fluctuations. 4 women used a consistently higher dose (>100mg) consistently throughout the 28 days. Two women who reported low daily doses of NRT (approximately 25mg) discontinued use within 3-4 days. Four women increased their daily NRT dose within the first seven days of initiating use, which may have been in response to cravings or advice from their stop smoking advisor.
