# Supplementary material for: The Relationship Between Reported Daily Nicotine Dose from NRT and Daily Cigarette Consumption in Pregnant Women Who Smoke in an Observational Cohort Study
Source: Nicotine Tob Res. 2023 Aug 3;26(2):212–9. doi: 10.1093/ntr/ntad140 (PMC10803113; doi:10.1093/ntr/ntad140)
Supplement: ntad140_suppl_Supplementary_File_S3 [file ntad140_suppl_supplementary_file_s3.pdf]

Supplementary file 3

Table S3. Multilevel repeated measures model of the relationship between Daily NRT dose and cigarettes per day

	**Regression Coefficient (95% CI)**
**Fixed effects**	
Daily NRT dose spline 1	-0.07 (-0.09 - -0.04)**
Daily NRT dose spline 2	0.02 (-0.01 – 0.04)
Daily NRT dose spline 3	-0.03 (-0.04 - -0.02)*
Daily NRT dose spline 4	-0.01 (-0.02 - -0.00)**
**Random effects**	**Variance (standard error) (95% CI)**
Participant	4.15 (SE 1.23) (0.21 – 0.41)

*p<0.05, **p<0.001

(Model controls for autocorrelation)
